# Spontaneous intake of essential oils after a negative postnatal experience has long-term effects on blood transcriptome in chickens

**DOI:** 10.1038/s41598-020-77732-5

**Published:** 2020-11-26

**Authors:** Aline Foury, Anne Collin, Jean-Christophe Helbling, Christine Leterrier, Marie-Pierre Moisan, Laurence A. Guilloteau

**Affiliations:** 1 INRAE, Bordeaux INP, NutriNeuro, UMR 1286, University Bordeaux, 33076 Bordeaux, France; 2 INRAE, Université de Tours, BOA, 37380 Nouzilly, France; 3 INRAE, CNRS, IFCE, Université de Tours, PRC, Nouzilly, France

**Keywords:** Physiology, Health care

## Abstract

Chicks subjected to early stressful factors could develop long-lasting effects on their performances, welfare and health. Free access to essential oils (EO) in poultry farming could mitigate these effects and potentially reduce use of antimicrobial drugs. This study on chicken analyzed long-lasting effects of post-hatch adverse conditions (Delayed group), and the impact of EO intake on blood physiological parameters and transcriptome. Half of the Control and Delayed groups had free access to EO, while the other half had only water for the first 13 days post-hatching. Blood analyses of metabolites, inflammation and oxidative stress biomarkers, and mRNA expression showed sex differences. Long-lasting effects of postnatal experience and EO intake persisted in blood transcriptome at D34. The early adverse conditions modified 68 genes in males and 83 genes in females. In Delayed males six transcription factors were over-represented (NFE2L2, MEF2A, FOXI1, Foxd3, Sox2 and TEAD1). In females only one factor was over-represented (PLAG1) and four under-represented (NFIL3, Foxd3, ESR2 and TAL1::TCF3). The genes showing modified expression are involved in oxidative stress, growth, bone metabolism and reproduction. Remarkably, spontaneous EO intake restored the expression levels of some genes affected by the postnatal adverse conditions suggesting a mitigating effect of EO intake.

## Introduction

In poultry production systems, broiler chicks can be exposed to various stressful factors in the hatchery, during transportation to rearing houses and in their first days of life. These early-life stresses have long-lasting effects on their performances, but also on welfare and health^1,2^ often requiring the use of antibiotics. Alternatives to antibiotics treatment used in animal production is a field of intense research, driven by the emergence of resistance to bacteria and the societal demand for more sustainable rearing practices and veterinary medicines. An appealing alternative to antibiotics/chemical antimicrobial drugs is the use of specific plants or substrates with medicinal properties to control or to prevent diseases. In particular, essential oils (EO) extracted from aromatic plants are known to offer multi-functional medicinal properties including antimicrobial, antioxidant, anti-inflammatory, immune and nervous regulatory properties^3–6^. These regulatory properties are linked to the terpenoids (monoterpenes, sesquiterpenes) and aromatic compounds present in EO. Phenols, alcohols and ketones have also been reported to have an antibacterial action^7^. In chickens, EO have been employed as feed additives to promote growth and health^8,9^. However, in most studies, the EO were included in the feed of the animal and their ability to mitigate long-term effects of early-life stresses was never analyzed.

In a previous study, we developed an experimental model reproducing adverse perinatal conditions in chicks including the deprivation of feed and water, unpredictable shaking in transportation boxes and temperature changes 24 h after hatching, summarized as “negative postnatal experience”. These conditions resulted in reduced growth up to 34 days of age^10^, fecal-specific odors detectable by rats^11^ and a fecal metabolomic signature highlighting persistent differences in adaptive response, energy metabolism and microbiota composition compared to optimal conditions^12^. Interestingly, we reported that chicks provided with EO independently of their feed were able to self-select which EO they consumed in response to their postnatal experience and that this EO intake mitigated some detrimental effects on growth parameters induced by this negative postnatal experience^10^.

To complete these data, we examined whether the spontaneous intake of EO would mitigate the long-lasting consequences of negative postnatal experience on health parameters. More precisely, we hypothesized that the negative postnatal experience would alter blood physiological parameters such as metabolites, inflammation and oxidative stress biomarkers and reveal a specific transcriptomic signature in blood cells, but also that EO intake may restore these physiological parameters. We focused on gene expression profiling to assess health status because it is a non-invasive approach that is increasingly used to obtain a general, non-biased view of the biological pathways that are affected by a given condition. This approach has been used to assess the effects of early life stress in humans^13^ and also in animals^14^ including laying hens^15^ but not in broiler chickens.

Based on our previous study, three EO were chosen for their complementary properties to control microbial infections, reduce stress response, and improve digestive and immune system functions. Among the numerous biological activities of Cardamom EO, antioxidant, antispasmodic, anti-inflammatory, gastroprotective and antibacterial properties have been reported^16–18^. Marjoram EO demonstrates a variety of biological activities including a hepatoprotective role^19^, and lemon verbena EO has been shown to have analgesic, anti-inflammatory, sedative, and digestive properties^20^.

Thus, the objectives of the present study were i) to characterize the immediate and long-lasting effects of a negative postnatal experience on blood physiological parameters and the long-lasting effects on whole gene expression in blood cells of fast-growing broiler chickens and ii) to analyze the effects of EO intake on blood metabolites, inflammatory and redox statuses and mRNA expression of a selection of genes in chicks exposed or not to this negative postnatal experience.

## Results

In this section, to facilitate the reading, the postnatal negative experience is called “delayed placement” and the group concerned as the “Delayed group”.

### Immediate effects of delayed placement on physiological parameters of blood

**Figure 1:**
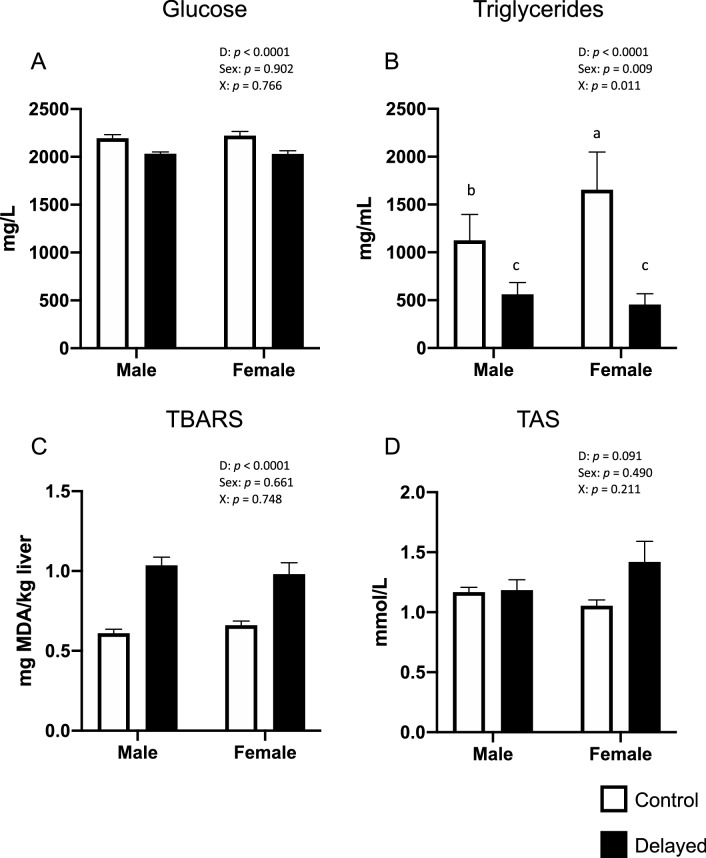
Effects of delayed placement and sex on metabolic and physiological parameters of plasma from one-day-old chicks: (**A**) glucose concentration; (**B**) triglyceride concentration (**C**) Thiobarbituric acid reactive substances (TBARS) and (**D**) Total Antioxidant Status (TAS). Bars represent the mean ± SEM of values measured in different groups. Two-way ANOVA results are shown for each parameter. D = delayed effect, X = interaction between delayed placement and sex effects.

The chicks of the Delayed group demonstrated a significant immediate effect on metabolism shown by a decrease in plasma glucose and triglyceride concentrations (p < 0.0001 for both) and on the redox balance shown by a significant increase in thiobarbituric acid reactive substances (TBARS; p < 0.001) in the liver, and as a tendency there was a rise in blood total antioxidant status (TAS; p = 0.091) (Fig. 1). A significant sex effect (p = 0.009) and interaction (p = 0.011) was found for triglycerides only.

### Long-lasting effects of delayed placement and EO intake on physiological parameters of blood

Metabolite plasma concentrations and redox balance differed significantly between males and females at D34. Triglyceride concentrations were higher in males than in females, whereas uric acid, TAS, ferric reducing ability of plasma (FRAP) and haptoglobin-like activities were higher in females than in males (Table 1).

**Table 1:** Metabolites and redox balance in blood of 34-day-old broiler chickens.

Physiological parameters	Male	Female	*p*-value
Glucose (g/L)	2.37 ± 0.04^a^	2.36 ± 0.05^a^	0.804
Triglycerides (mg/L)	0.80 ± 0.07^a^	0.62 ± 0.03^b^	**0.023**
Uric acid (mg/L)	56.57 ± 2.56^a^	66.34 ± 3.77^b^	**0.041**
TAS (mmol/L)	0.80 ± 0.02^a^	0.96 ± 0.04^b^	**0.001**
FRAP (µmol/L)	705.27 ± 22.53^a^	800.45 ± 24.80^b^	**0.007**
SOD (%)	77.79 ± 0.02 ^a^	76.01 ± 0.02 ^a^	0.439
TBARS (nm MDA/mL)	0.60 ± 0.02 ^a^	0.60 ± 0.03 ^a^	0.952
Haptoglobin-like (mg/mL)	0.18 ± 0.01^a^	0.22 ± 0.01^b^	**0.002**

Data are presented as mean ± SEM. Different letters correspond to significant differences (*p* < 0.05) between males and females (ANOVA).

**Figure 2:**
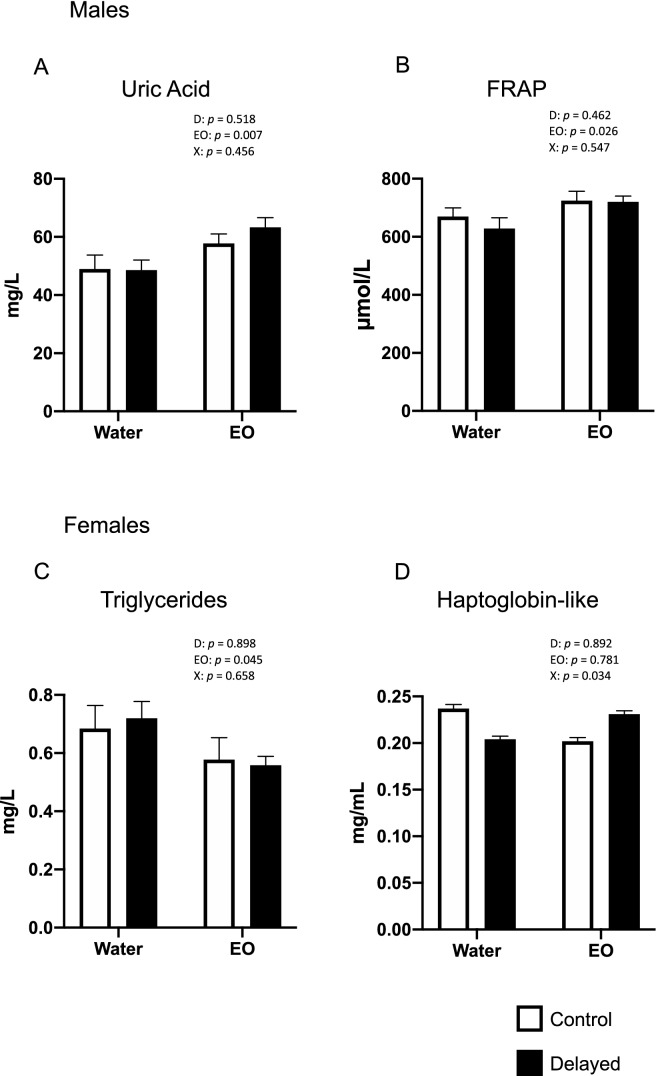
Effects of delayed placement and EO intake on physiological parameters of blood in 34-day-old male and female chickens: (**A**) urid acid concentration; (**B**) FRAP activity (**C**) triglyceride concentration and (**D**) haptoglobin-like. The bars represent the mean ± SEM of values measured in different groups. Two-way ANOVA results are shown for each parameter. D = delayed effect, EO = essential oil, X = interaction between delayed placement and EO effects.

At D34 in males, we observed no interaction between EO intake and the delayed placement after hatching and no delayed placement effects. However, EO intake had significant effects on two physiological parameters, namely uric acid and FRAP activity, which were increased with EO intake (Fig. 2A,B). At D34 in females, an interaction between EO intake and the delayed treatment was observed on haptoglobin-like activity (p = 0.034), but without significant differences between groups (Fig. 2D). No effect of the delayed treatment was observed, but triglyceride concentration was decreased with EO intake independently of the postnatal experience (Fig. 2C).

### Long-lasting effects of delayed placement on blood transcriptome in broiler chickens

**Figure 3:**
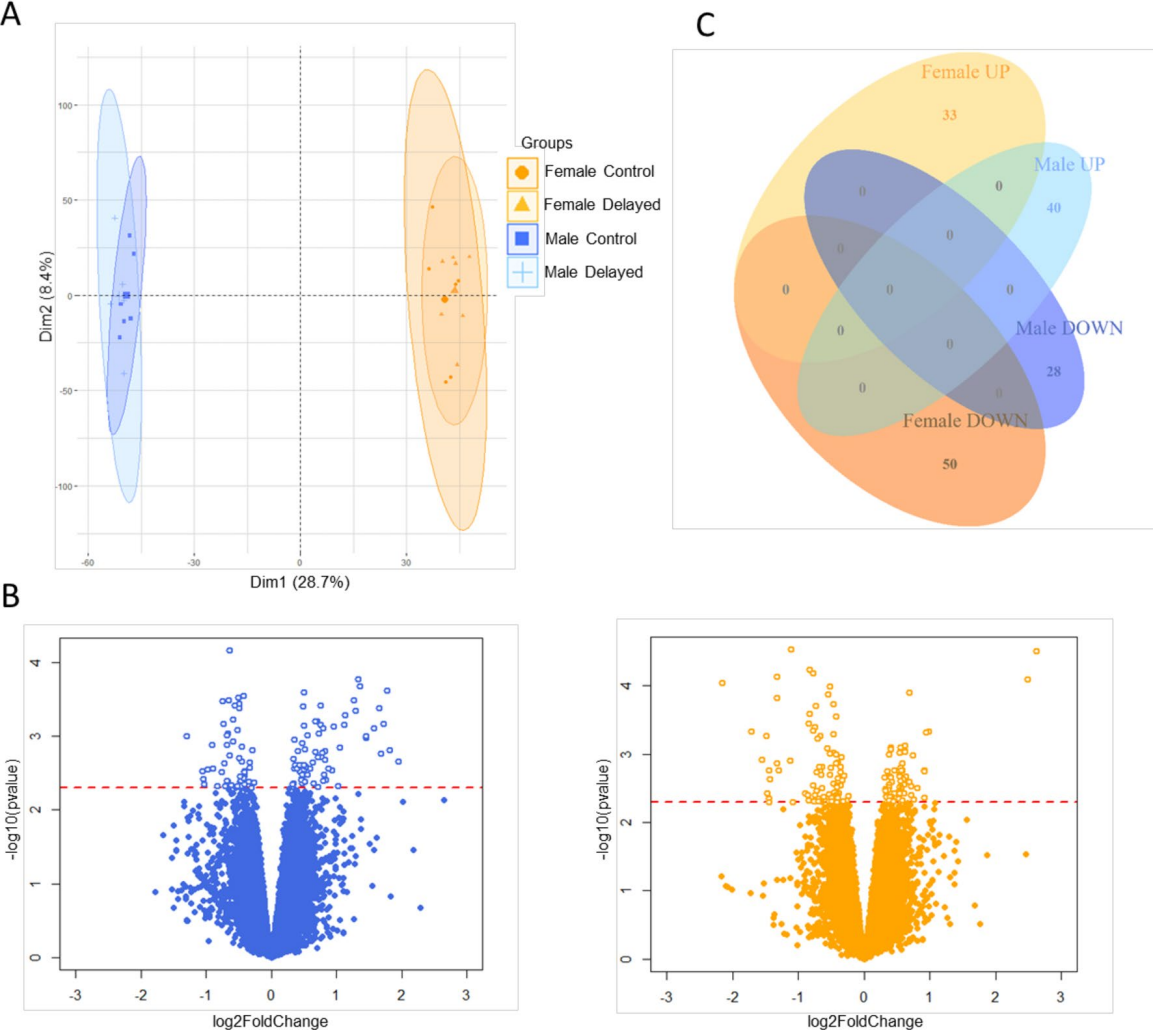
Microarray analysis of blood cells from 34-day-old chickens after delayed placement. Principal Components Analysis (PCA) conducted on microarray gene expression (normalized data), projection of individuals from each group and sex on the first two dimensions of the PCA (**A**). Volcano plots in male (blue) and female (orange) using –log10(non-adjusted p-value) on y-axis and log2(Delayed vs. Control chickens Fold Change) on x-axis. The red dotted line corresponds to a non-adjusted p-value = 0.005. Genes selected for further analyses are above this line (**B**). Venn diagram showing the number of differentially expressed genes between Delayed and Control groups for each sex and the lack of overlap between sexes (zero genes in common). UP = genes upregulated for Delayed chickens, DOWN = genes downregulated for Delayed chickens (**C**).

Microarray gene expression profiling was performed on blood cells from chickens at D34 in the Delayed and Control groups that had access to water only, to obtain a global biological signature of the long-lasting effects of the delayed placement. Microarrays consisted of 60,000 oligonucleotide probes, representing 12,349 annotated genes, i.e. 80% of the *Gallus gallus* genome. A principal component analysis of gene expression for each individual showed a strong sex effect (Fig. 3A), therefore sexes were analyzed separately thereafter. No genes were found to be expressed differentially when using an adjusted p-value < 0.05. A non-adjusted p-value of p < 0.005 was thus used (Fig. 3B Volcano plots). This analysis enabled 68 differentially regulated genes in males (40 up-regulated and 28 down-regulated in Delayed compared to Control group) and 83 differentially regulated genes to be detected in females (33 up-regulated and 50 down-regulated in Delayed compared to Control group). None of the differentially expressed genes were shared by males and females (Fig. 3C Venn diagram). The lists of differentially expressed genes are provided in Tables S1 (males) and S2 (females) in the supplementary files.

**Figure 4:**
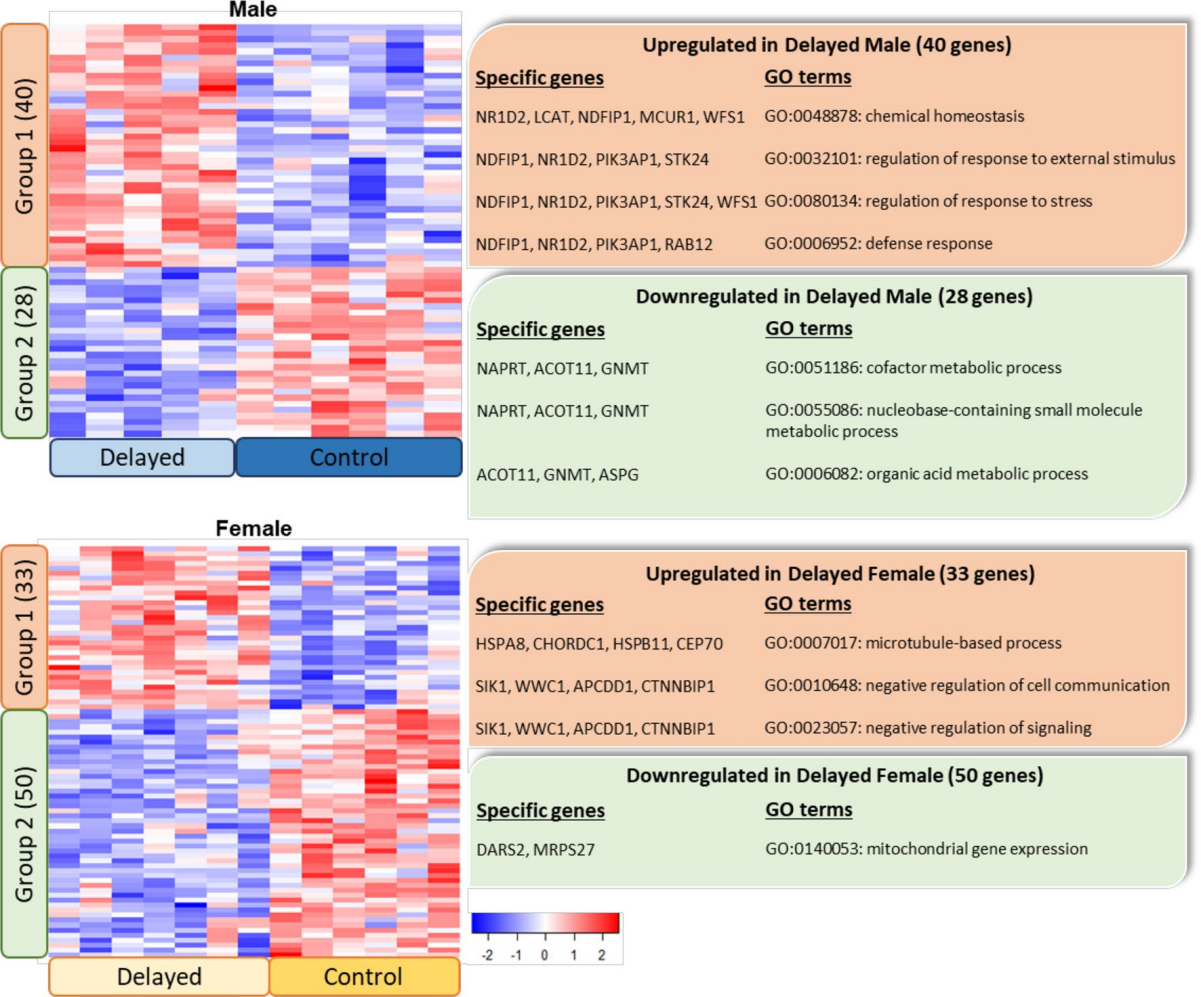
Heatmaps displaying 68 and 83 differentially expressed genes among Delayed and Control groups in males and females respectively. Enriched GO terms (non-adjusted p-value < 0.05) and genes are shown on the right for each group. The p-values of GO terms were determined on the WebGestalt website.

The Gene Ontology (GO) analysis of the differentially expressed genes for each sex indicated modification of broad biological processes (Fig. 4). Interestingly, in Delayed males the GO terms are related to an increase in response to stress and to external stimuli and in defense response and down-regulation of metabolic processes. In Delayed females, the GO terms suggest microtubule-based processes were modified, which would impact cell communication and signaling and decrease in mitochondrial gene expression.

**Figure 5:**
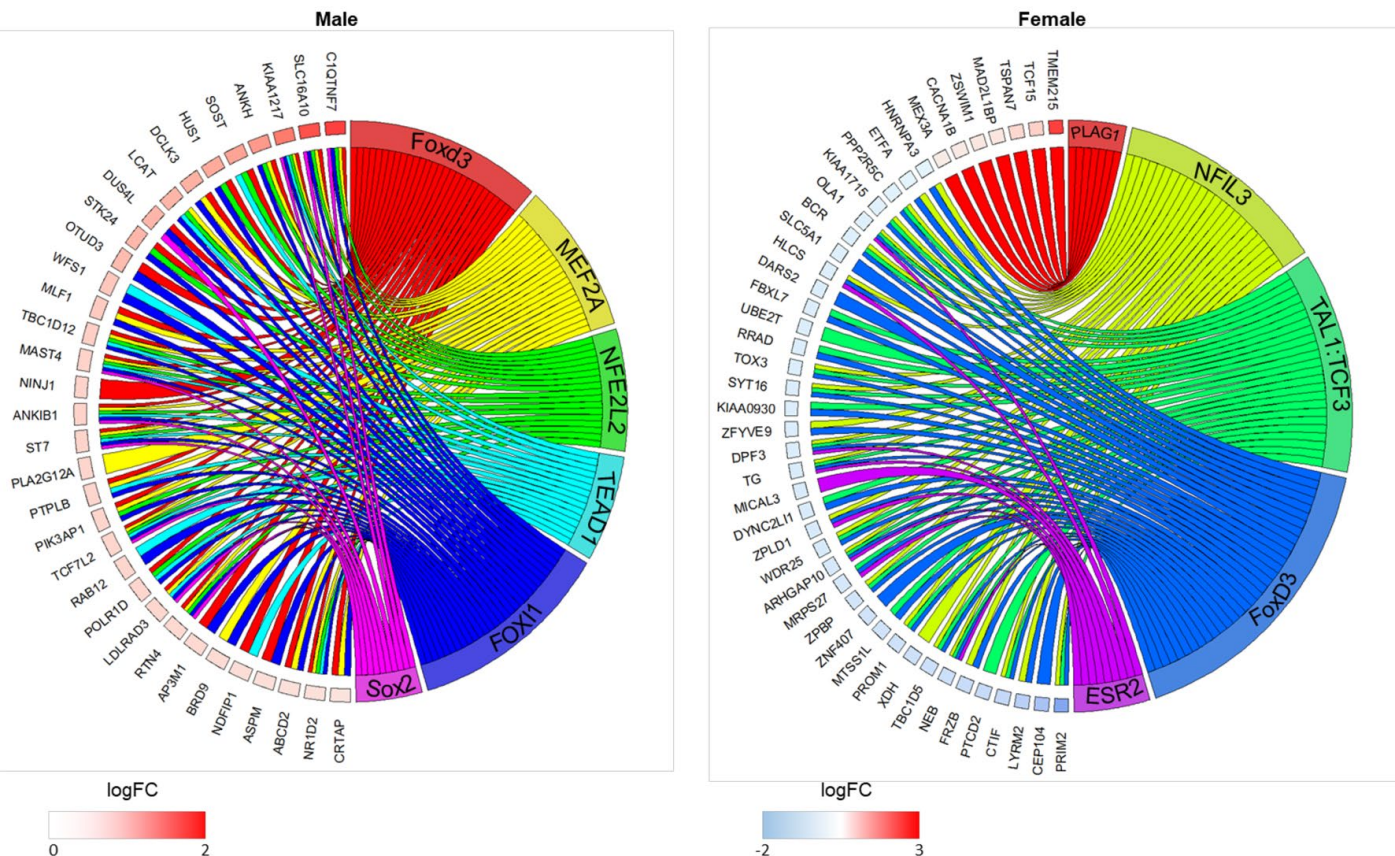
Circos plots (GOPlot R package) of over-represented conserved transcription factors (on the right side) and corresponding target genes (left side) enriched in Delayed compared to Control chickens for each sex. Up-regulated and down-regulated genes are indicated by a red and blue square, respectively. LogFC = log2 Fold Change. Significant over- or under-represented transcription factors were detected using oPOSSUM software.

To investigate the regulatory pathways that drive the expression of the differentially expressed genes we used the “single site” analysis of oPOSSUM software to detect over-represented conserved transcription factor binding sites in the set of differentially expressed genes for each sex. The transcription factors significantly over-represented in the Delayed compared to Control groups for each sex together with the corresponding target genes are shown in Fig. 5. In males, in the Delayed group no transcription factors were found significantly under-represented, but six transcription factors were over-represented, Foxd3, FOXI1, MEF2A, NFE2L2, Sox2 and TEAD1. In females, PLAG1 was the only transcription factor over-represented in the Delayed group. The other transcription factors NFIL3, ESR2, TAL1::TCF3 and Foxd3 were under-represented in the Delayed group. It should be noted that Foxd3 was over-represented in Delayed males, but under-represented in Delayed females.

### Long-lasting effects of EO intake on blood gene expression in broiler chickens after the delayed placement

To evaluate the effect of chick’s EO intake on the blood transcriptome according to their postnatal experience, we performed quantitative PCR (qPCR) on a selection of genes based on the microarray results. We focused on the oxidative stress pathway identified in males (represented by NFE2L2 and MEF2A transcription factors) and on the PLAG1 pathway identified in females by selecting gene targets of either NFE2L2 or MEF2A (n = 18) and targets of PLAG1 (n = 5) according to oPOSSUM. To complete the gene selection, eleven genes involved in oxidative stress or inflammation not tested on the microarray were also analyzed and called “oxidative stress” or “inflammation” genes respectively. Additionally, other genes which were significantly differentially expressed between Control and Delayed groups in males (n = 10) and females (n = 4) were selected and called “other responsive genes”. The list of these 48 selected genes, their primer sequences used for quantitative real time PCR and their associated signaling pathways are presented in Table S3.

Quantitative PCR were conducted on blood cell cDNA from D34 chickens tested on the microarray analysis (Control and Delayed groups) and also those tested for EO or water intake (EO and W groups).

**Figure 6:**
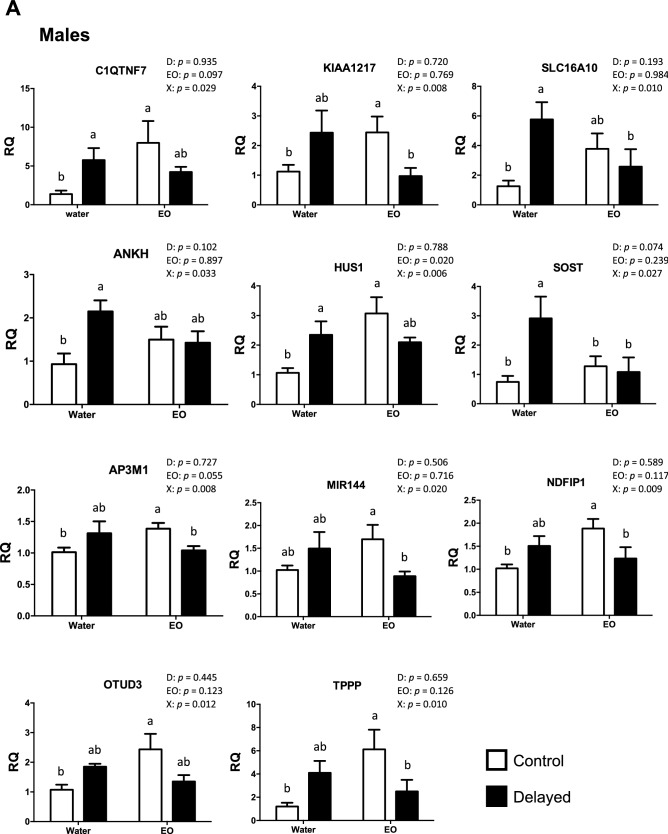

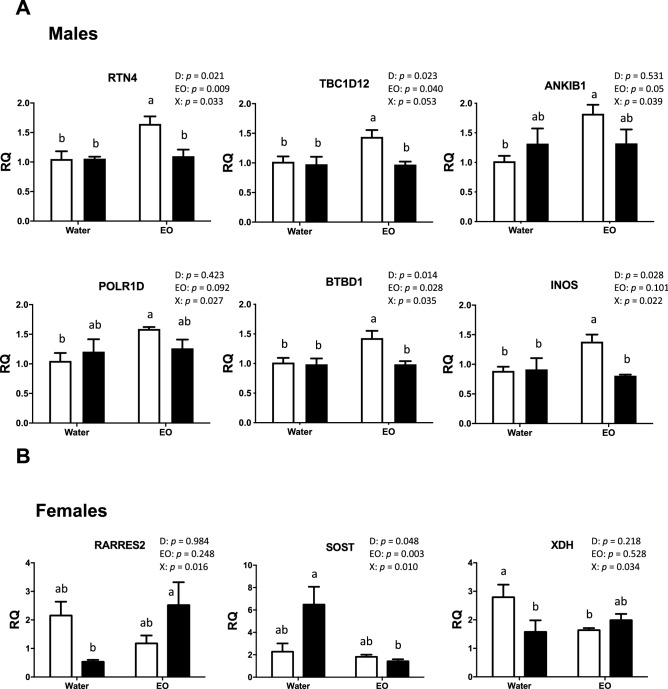
Effects of EO intake on blood-cell gene expression in 34-day-old male (**A**) and female (**B**) chickens after the delayed placement (qPCR analyses). Bars represent the mean ± SEM of values measured in different groups. Different letters between bar-plots indicate significant or tendency differences (p < 0.05 or p < 0.10) between groups detected by post-hoc analyses following two-way ANOVA.

Significant interactions of gene expression were found between the delayed placement and the chickens’ intake of EO. Among all the genes tested, a significant interaction or tendency was found in 19 out of 33 genes in males and in three out of 15 genes in females. In males provided with water only, a significant effect of the delayed placement on gene expression was confirmed in five genes involved in oxidative stress (C1QTNF7, SLC16A10, ANKH, HUS1 and SOST), but in only one gene (XDH) in water-only females (Fig. 6A,B). A significant effect (or tendency) of the delayed placement was also confirmed for the expression of four other genes in males and five genes in females, independently of the EO intake (Table 2). These genes were significantly differentially regulated on the microarray as “other responsive genes” (ASPM, BRD9 and PTPLB) in males, or involved in oxidative stress (SOD3) or inflammation (COX2 and PIT54) and in the PLAG1 pathway (MEX3A, TMEM125 and ZSWIM1) in females.

**Table 2:** Placement and EO effects on blood RNA expression in 34-day-old chickens after a negative postnatal experience.

Gene	Placement		Placement p-value	Treatment		Treatment p-value	Placement x Treatment interaction p-value
	Control	Delayed		Water	EO		
**Males**							
BRD9	1.26 ± 0.14	0.89 ± 0.11	0.050^†^	0.96 ± 0.12	1.19 ± 0.14	0.195	0.388
PTPLB	1.13 ± 0.18	0.64 ± 0.13	0.055^†^	1.02 ± 0.13	0.81 ± 0.19	0.493	0.364
ASPM	1.50 ± 0.19	1.00 ± 0.11	0.023*	1.05 ± 0.14	1.50 ± 0.18	0.038*	0.174
COX2	0.82 ± 0.12	0.52 ± 0.11	0.052^†^	0.97 ± 0.08	0.44 ± 0.10	0.001*	0.580
PIK3AP1	1.34 ± 0.15	1.11 ± 0.09	0.135	1.01 ± 0.08	1.44 ± 0.14	0.011*	0.255
DUS4L	1.46 ± 0.18	1.34 ± 0.16	0.633	1.18 ± 0.19	1.63 ± 0.13	0.086^†^	0.615
**Females**							
MEX3A	3.10 ± 0.86	12.30 ± 3.95	0.038*	7.74 ± 2.23	7.65 ± 4.01	0.673	0.336
TMEM125	1.05 ± 0.31	3.63 ± 0.92	0.010*	2.05 ± 0.57	2.81 ± 1.06	0.225	0.073
ZSWIM1	1.51 ± 0.16	2.34 ± 0.35	0.035*	1.67 ± 0.19	2.19 ± 0.37	0.127	0.668
PIT54	1.18 ± 0.63	4.58 ± 1.11	0.052^†^	4.23 ± 1.19	1.91 ± 0.91	0.268	0.911
SOD3	2.70 ± 1.25	10.29 ± 3.01	0.080^†^	8.61 ± 2.87	5.10 ± 1.57	0.417	0.764

q PCR data (mean ± SEM) were only shown for significant gene expression without significant interaction between the two factors (ANOVA).

* p < 0.05 considered significant and ^†^ 0.05 < *p* < 0.1 considered to be a tendency.

Interestingly, the Delayed group showed a clear favorable effect of EO intake for the SOST gene in males and females, for the SLC16A10 gene in males, and for the RARRES2 gene in females (Fig. 6A,B). Indeed, for these genes, EO intake in the Delayed group significantly restored the gene expression level observed in the Control group supplied with water only. Unexpectedly, the intake of EO significantly modified the gene expression in the Control group for 13 genes in males and for one gene (XDH) in females. These genes were involved in oxidative stress (C1QTNF7, KIAA1217, HUS1, RTN4, TBC1D12, ANKIB1, POLR1D and INOS) or qualified as “other responsive genes” in the microarray analysis (AP3M1, NDFIP1, OTUD3, TPPP, BTBD1 and XDH) (Table S3, Fig. 6A,B). A significant effect of EO intake was also observed in males on the expression of ASPM, COX2 and PIK3AP1 genes, whatever the postnatal experience (Table 2).

## Discussion

This study presents immediate and long-lasting effects of a delayed placement of chickens considered as a negative postnatal experience, mimicking early-life stresses encountered in poultry production, and the potential beneficial effects of EO intake on blood physiological parameters and gene expression profiling of chicken blood cells. In a previous study, we showed that chicks spontaneously chose to consume EO or not, according to their postnatal experience when different EO were presented individually^10^.

In this study, we detected significant differences between sexes for several blood parameters. Triglyceride concentrations were higher in females than in males after hatching, but this was reversed at D34 indicating lower lipid metabolism in females at that age. At D34, higher anti-oxidative activity (uric acid, TAS and FRAP) and higher haptoglobin-like activity were observed in females than in males.

In both sexes, the negative postnatal experience resulted in an immediate decrease in blood glucose (typically around 2 g/L in control broiler chickens as commonly observed in this species^21^) and triglycerides associated with an increase in blood and liver lipid peroxidation (TBARS). This indicates that chicks exposed to these postnatal conditions adapted metabolically and suggests that they used the nutrients present in the internalized yolk sac^22^. Such a decrease in glycaemia was not observed in a previous study where chicks were subjected to early post-hatch delayed feeding^23^ suggesting that in our study, this decrease could be the result of the combined effects of delayed feeding, variations in ambient temperatures and transport box shaking that may increase chick activity within the box. It could also be linked to a decrease in insulinemia as reported by Bigot et al.^23^, not measured in the present experiment. This adaptive metabolic profile was present in fecal metabolome of chicks for at least for 13 days after the delayed placement, suggesting that the adaptive changes in metabolism due to the postnatal experience persisted^12^, but no such effect was observed on blood physiological parameters at D34 in the present study.

An in-depth view of the long-lasting effects of the negative postnatal experience on the physiology of the chickens was investigated by analyzing gene expression profiles of whole blood at D34 using microarray and qPCR. Very strong sex differences in the response to early external stimuli were observed at D34 in the blood gene expression profiles for which overall sex differences explain 28.7% of the variability in gene expression and from the microarray analysis none of the differentially expressed genes were shared between males and females.

Other studies have reported sex differences in chickens regarding stress response, including early stress, on behaviour, sex hormones and hypothalamic gene expression^15,24,25^. The results of the GO analysis were limited by the low number of genes differentially expressed between groups. Nevertheless, it is remarkable that the terms ‘response to external stimuli’, ‘response to stress’ and ‘defense response’ were enriched in Delayed males. The oPOSSUM analysis provided more precise data on the pathway involved. In particular, the negative postnatal experience of chicks induced changes in the expression of genes belonging to the oxidative stress pathway (as represented by the transcription factors NFE2L2 and MEF2A) in males and to a much lower extent in females. Using qPCR, seven gene targets of these two transcription factors were confirmed to be differentially regulated (BRD9, PTPLB, C1QTNF7, SLC16A10, ANKH, HUS1 and SOST) in Delayed males and three genes involved in oxidative stress response (XDH, SOD3 and PIT54) were also differentially regulated in the Delayed group in females. MEF2A is also known to influence muscle development and has been described in chicken^26^. Thus the MEF2A pathway may be activated to counter the observed decrease in *P. major* muscle yield observed in the Delayed chick group^10^.

Besides oxidative stress, a number of the differentially regulated genes in males have interesting properties, such as the up-regulated genes related to bone metabolism: ANKH has a role in osteogenic differentiation of bone marrow stromal cells and is a vascular calcification inhibitor^27,28^ and SOST is a negative regulator of bone growth^29,30^. Moreover, the transcription factor TEAD1 over-represented in the Delayed males supports an effect of delayed placement on bone metabolism by regulating organ size and skeletal muscle mass^31^. In line with this, SLC16A10 gene over-represented in the Delayed males is a transporter gene belonging to a family of transporters involved in energy metabolism, including thyroid hormone metabolism and possibly skeletal development^32^.

In females, the PLAG1 pathway was the only pathway over-represented following the negative postnatal experience. PLAG1 is a regulator of growth and reproduction^33^ and associated with variability in egg production in laying hens^34^. Through qPCR, three genes targets of PLAG1 (MEX3A, TMEM215, ZSWIM1) were shown to be up-regulated in the female Delayed group. This is interesting in view of the reduced growth observed in the chicks of the Delayed group. Some other significant pathways were under-represented following the negative postnatal experience and involved gene targets of NFIL3, Foxd3 and ESR2. NFIL3 is involved in the circadian rhythm in the chick’s pineal gland and is essential as a regulator of the immune response^35^. ESR2 plays a central role in folliculogenesis and therefore in reproduction^36^. Foxd3 is important in embryogenesis being involved in regulating the lineage choice between neural crest-derived glial cells^37^ and in immunity by regulating IL-10-positive B cells^38^. Foxd3 under-represented in Delayed females was in contrast over-represented in Delayed males. This may be related to the higher immune resistance of female compared to male chicken as reported in a large study on mortality following infectious ^39^.

Remarkably, the spontaneous intake of these EO by chicks for 12 days after hatching had long-lasting beneficial effects on the expression of several of these genes modified by the negative postnatal experience. The expression of SOST (both in males and females), SLC16A10 (only in males) and RARRES2 (only in females) genes was restored to the expression observed in the Control group when chickens had access to EO. Interestingly, RARRES2 is an adipokine that plays a major role in the regulation of metabolic and reproductive processes recently studied in the chicken^40^, in line with the list of PLAG1 gene targets, and is also a chemokine for leucocyte populations and participates in antimicrobial activity.

In our study, the beneficial role of EO intake after the negative postnatal experience could be to regulate the growth and bone metabolism in male and female chicken, and also the energy metabolism and reproduction in females.

Surprisingly, EO intake by control chickens modified the expression of 17 genes, 16 in males and one in females. Most of them are involved in oxidative stress, immune regulation and microtubule network integrity. The majority of the genes studied and up-regulated in the Control group were different to those up-regulated in the Delayed group suggesting that the reasons why chicks ingested EO differed between these two placement conditions.

To our knowledge, this is the first study in chickens to show long-lasting effects of spontaneous intake of EO on gene expression, especially changing some blood expression patterns affected by the chick’s post-hatch experience. Further studies are needed to improve the understanding of these effects on blood transcriptome, in tissues of the liver and gut for example and on gut microbiota which could be considerably modified by the antimicrobial properties of EO. The self-medication behaviour in domestic animals warrants further investigation, especially regarding infectious challenges. It could be greatly beneficial for animals like chickens raised in large groups to be allowed to manage their health and welfare individually through free access to EO, hence limiting the use of antibiotics in the context of the One Health concept.

## Methods

### Essential oils (EO)

Cardamom (*Elettaria cardamomum*) (1480CQ, batch S12A, Herbes et Traditions, Comines, France), marjoram (*Origanum majorana*) CT thujanol (2507CQ, batch S12D, Herbes et Traditions), and lemon verbena (*Lippia citriodora*) (FLE094, batch H181013MA, Florihana, Caussols, France) were chosen based on the properties of their major components and on our previous study which showed spontaneous intake and long-term effects after a negative postnatal experience in chicks^10^. Each EO was diluted in water (0.001%), mixed and shaken vigorously before being made available in a drinking bottle. The main components obtained by gas chromatography coupled to mass spectrometry for each EO are listed in Table 3.

**Table 3:** Essential oils composition (from [10]).

Compound	Cardamom ***Elettaria cardamomum***		Marjoram ***Origanum majorana*** CT thujanol		Verbena ***Lippia citriodora***	
	Specification (%)	Relative content (%)^a^	Specification (%)	Relative content (%)^b^	Specification (%)	Relative content (%)^c^
Monoterpenes	6—12	13	30	40	5—15	29
Sesquiterpenes				3	18—26	24.5
Monoterpenols	3—6	5	40 – 50 (20 thujanol)	50 (25 thujanol)	3—15	2
Esters	39—51	36		2		
Oxides	27—35	34			< 7	5
Aldehydes					20—40	24

^a^1480CQ, batch S12A, Herbes et Traditions, Comines, France.

^b^2507CQ, batch S12D, Herbes et Traditions.

^c^FLE094, batch H181013MA, Florihana, Caussols, France.

### Experimental design

A model of postnatal negative experience was previously developed to analyze the consequences of this experience over the whole growing period of broiler chickens^10^. This model involved reproducing suboptimal transportation conditions for chicks after hatching. The chicks (Hubbard Classic, Quintin, France) were either placed immediately in pens in the rearing facility after their removal from the incubator (Control group, C, n = 192) or they were removed and subjected to 24 h of negative experience before being placed in pens (Delayed group, D, n = 192). This delayed group was deprived of both food and water and subjected to irregular movement and various room temperatures^10^. Half of the chicks (six pens each for C and D groups) had access to water only in four bottles (W–C and W-D groups). Besides feed and water supplies, the other half of the chicks had ad libitum access to the three EO (EO-C or EO-D) from D1 until D12 post-hatching. One bottle containing water, and the three others each containing one of the EO, were placed in each pen. Chicks were reared at the Experimental Poultry Facility (PEAT, INRAE, 2018, 37,380 Nouzilly, France, 10.15454/1.5572326250887292E12) under standard temperature and light conditions with ad libitum access to water and with a wire mesh platform and a perch for environmental enrichment. At D13, the chickens were transferred to another poultry building for the growth phase until D34. They had ad libitum access to feed without anticoccidial drugs. They were fed with a standard starting diet (metabolizable energy = 12.8 MJ/kg, crude protein = 22%) until D19 and then a rearing diet from D19 to D34.

Physiological analyses were performed at D1 (first experiment), and D34 post hatching (second experiment) on plasma and liver samples from 12 chickens (6 males and 6 females) per condition (2 conditions × 2 experiments × 12 chickens = 48 chickens). Transcriptomic analysis was performed at D34 on blood samples from the same chickens as those sampled for physiological parameters. All procedures used in these experiments were approved by the local ethics committee (Comité d’Ethique en Expérimentation Animale Val de Loire, Tours, France; permission no 01730.02 and 2015070815347034v2 (APAFIS#1082) and carried out in accordance with current European legislation (EU Directive 2010/63/EU).

### Physiological parameters

Different metabolic, antioxidant and oxidative statuses and inflammation parameters were measured at D1 and D34 after hatching. Commercial kits (THERMO FISHER DIAGNOSTICS SAS, Courtaboeuf, France) were used to determine plasma glucose (g/L) (MG981780), uric acid (mg/L) (MG981788) and triglycerides (mg/L) (MG981786). Total plasma antioxidant activity was determined through Total antioxidant status (TAS) (mmol/L) (NX 2332, RANDOX LABORATOIRES, Roissy, France). Protocols were used in accordance with supplier instructions and adapted to the automated Thermo Scientific Arena 20XT photometric analyzer (THERMO FISHER DIAGNOSTICS). The Ferric reducing/antioxidant power (FRAP) was determined as described by Benzie and Strain^41^ and results expressed as µmol Trolox/L. Plasma superoxide dismutase (SOD) activity was measured with a commercial kit (19,160, Sigma-Aldrich Chemie GmbH, Buchs, Switzerland), using a microplate reader (TECAN infinite 200, Tecan Group Ltd, Männedorf, Switzerland) and expressed as an inhibition activity (%). Haptoglobin-like activity described as PIT54 in chicken^42^ was evaluated in plasma using a colorimetric assay (TP-801, Tridelta Development Ltd, Maynooth, Ireland) measuring inhibition of hemoglobin peroxidase activity and expressed as mg/mL. Lipid peroxidation was determined using spectrophotometry of thiobarbituric acid reacting substances (TBARS) as previously described by Lynch and Frei for the liver^43^, and adapted from Lin et al.^44^ for plasma TBARS.

### RNA extraction and gene expression analyses

#### RNA extraction

Blood was collected from chickens at D34 using 5 ml EDTA vacutainer tubes. Blood (v = 100 µL) was immediately suspended in 1 mL of Invitrogen TRIzol reagent (FISHER SCIENTIFIC, Illkirch-Graffenstaden, France) and vigorously shaken for 5 min on ice. The samples were kept at -80 °C until RNA extraction. Total RNA was extracted using TRIzol (FISHER SCIENTIFIC) following the manufacturer’s protocol modified in accordance with Desert et al.^45^. Ten µl of acetic acid 5 N were added with chloroform to reduce DNA contamination. The quality of total RNA was assessed using RNA Nano chips on a 2100 Bioanalyzer Instrument (Agilent, Waldbronn, Germany). All the samples had an RNA Integrity Number (RIN) score of > 8.0.

### Microarrays

Gene expression profiles were performed at the GeT‐TRiX facility (GenoToul, Génopole Toulouse, Toulouse, Midi-Pyrénées) using Agilent SurePrint G3 gallus_exp_microarray_SLagarrigue_8 × 60k_V2_july2012 microarrays (8 × 60 K, design 042,004) following the manufacturer's instructions. For each sample, Cyanine-3 (Cy3) labeled cRNA was prepared from 200 ng of total RNA using the One-Color Quick Amp Labeling kit (Agilent) in line with the manufacturer's instructions, followed by RNA clean-up using Agencourt RNAClean XP (Agencourt Bioscience Corporation, Beverly, Massachusetts). Dye incorporation and cRNA yield were checked using Dropsense 96 UV/VIS droplet reader (Trinean, Ghent, Belgium). Aliquots of 600 ng of Cy3-labelled cRNA were hybridized on the microarray slides following the manufacturer’s instructions. Immediately after washing, the slides were scanned on an Agilent G2505C Microarray Scanner using Agilent Scan Control A.8.5.1 software and the fluorescence signal was extracted using Agilent Feature Extraction software v10.10.1.1 with default parameters. Microarray data and the experimental details are available in the NCBI’s Gene Expression Omnibus^46^ and are accessible through GEO Series accession number GSE102358 (https://www.ncbi.nlm.nih.gov/geo/query/acc.cgi?acc=GSE102358).

### Real-time quantitative PCR (qPCR)

To avoid genomic DNA amplification, primer pairs were designed in two different exons (thus spanning an intron) using the Primer Express software (Applied Biosystems, THERMO FISHER DIAGNOSTICS). The sequences of primers used are provided in Table S3. The specificity of the PCR reaction was validated according to MIQE (Minimum Information for publication of Quantitative real time PCR Experiments) guidelines^47^. An aliquot of 2,000 ng of total RNA was reverse-transcribed in cDNA with Superscript III (Invitrogen, THERMO FISHER DIAGNOSTICS) and random hexamers in accordance with the manufacturer’s protocol. High throughput real time quantitative PCR was performed on the Biomark HD System (Fluidigm, Les Ullis, France) following the manufacturer’s protocol. The chip was placed into the IFC Controller, where 6.3 nl of Sample Mix and 0.7 nl of Assay Mix were mixed. Real-time PCR was performed on the Biomark System as follows: Thermal Mix at 50 °C, 2 min; 70 °C, 30 min; 25 °C, 10 min, UNG at 50 °C, 2 min, Hot Start at 95 °C, 10 min, PCR Cycle of 35 cycles at 95 °C, 15 s; 60 °C, 60 s and Melting curves (from 60 °C to 95 °C). Results were analyzed using Fluidigm Real-Time PCR Analysis software v.4.1.3. (Fluidigm) to control specific amplification for each primer, then the raw results of the qPCR were analyzed using GenEx software (MultiD analyses AB, Götegorg, Sweden) in order to choose the best reference gene to normalize mRNA expression and measure the relative expression of each gene between groups. HPRT was found to be the best reference gene in this experiment and was thus used for the normalization of gene expression.

### Statistical analyses

The effects of a negative postnatal experience and EO intake and their interaction on physiological responses and qPCR analyses were determined using 2-way analysis of variance (ANOVA) after having checked the normality of data distribution. When there was an interaction between variables, a Fisher (LSD) test was used to determine the statistical significance of the difference. Differences were considered significant when p-values < 0.05 and a tendency for p < 0.10. Analyses were performed using XLSTAT software (version 2015, Addinsoft, Paris, France).

Microarray data were analyzed using R^48^ and Bioconductor packages (www.bioconductor.org, v 3.0)^49^ as described in GEO accession GSE102358. Briefly, raw data (median of pixel intensity) were filtered, log2 transformed, corrected for bath effects (washing and labeling serials) and normalized using the quantile method^50^. Principal Component Analysis (PCA) of the normalized microarray data was used to create a factor map of individual global expression using R package FactoMineR^51^.

A model was fitted using the limma lmFit function^52^ considering sex as a blocking factor. A correction for multiple testing was then applied using the Benjamini–Hochberg procedure (BH)^53^ for the false discovery rate (FDR). Probes with FDR ≤ 0.05 were considered to be differentially expressed between conditions. For bioinformatic analyses we selected probes that displayed a non-adjusted p value of p < 0.005 as too few probes were found with a FDR ≤ 0.05. All differentially expressed genes, screened on conditions of showing a ≥ 20% difference in mean expression levels between samples from delayed and control conditions, and a non-adjusted p-value < 0.005, were subjected to GO classification [GO-BP (biological process)] to assign these genes to relevant GO Terms using WebGestalt website^54^. oPOSSUM website was used to determine the over-representation of transcription factor binding sites within the differentially expressed genes^55^.

## Supplementary information

Supplementary Information.
