# Supplementary material for: Spontaneous intake of essential oils after a negative postnatal experience has long-term effects on blood transcriptome in chickens
Source: Sci Rep. 2020 Nov 26;10:20702. doi: 10.1038/s41598-020-77732-5 (PMC7691513; doi:10.1038/s41598-020-77732-5)
Supplement: Supplementary file 1 — Supplementary Information. [file 41598_2020_77732_MOESM1_ESM.pdf]

Spontaneous intake of essential oils after a negative postnatal experience has long-term effects on blood transcriptome in chickens

Aline Foury[1], Anne Collin[2], Jean-Christophe Helbling[1], Christine Leterrier[3], Marie-Pierre Moisan[1]*, Laurence A. Guilloteau[2]*†

[1]University Bordeaux, INRAE, Bordeaux INP, NutriNeuro, UMR 1286, 33076, Bordeaux, France ; aline.foury@inrae.fr (AF) ; jean-christophe.helbling@inrae.fr (J-C H), marie-pierre.moisan@inrae.fr (M-P M)

[2]INRAE, Université de Tours, BOA, 37380 Nouzilly, France; anne.collin@inrae.fr (AC) ; laurence.guilloteau@inrae.fr (LG)

[3]INRAE, CNRS, IFCE, Université de Tours, PRC, Nouzilly, France; christine.leterrier@inrae.fr (CL)

* Both authors contributed equally to this work.

† Correspondence: laurence.guilloteau@inrae.fr; Tel.: +33-247427946

Table S1. Differentially expressed genes up- and down-regulated in male chickens

Gene symbol	Fold Change	Gene name
C1QTNF7	3,87	C1q and TNF related protein 7
SLC16A10	3,54	solute carrier family 16 member 10
KIAA1217	2,73	enhancer trap locus 4
TPPP	2,52	tubulin polymerization promoting protein
ANKH	2,20	ANKH inorganic pyrophosphate transport regulator
SOST	2,04	sclerostin
HUS1	1,93	HUS1 checkpoint clamp component
DCLK3	1,84	doublecortin-like kinase 3
LCAT	1,78	lecithin cholesterol acyltransferase
DUS4L	1,77	dihydrouridine synthase 4-like
STK24	1,76	serine/threonine kinase 24
IMPA2	1,75	inositol monophosphatase 2
ZNF555	1,74	zinc finger protein 555
OTUD3	1,70	OTU deubiquitinase 3
FAM103A1	1,66	family with sequence similarity 103 member A1
MILR1	1,60	mast cell immunoglobulin like receptor 1
WFS1	1,55	wolframin ER transmembrane glycoprotein
MLF1	1,51	myeloid leukemia factor 1
TBC1D12	1,46	TBC1 domain family, member 12
CCDC90A	1,45	mitochondrial calcium uniporter regulator 1
MAST4	1,42	microtubule associated serine/threonine kinase family member 4
NINJ1	1,42	ninjurin 1
ANKIB1	1,42	ankyrin repeat and IBR domain containing 1
ST7	1,41	suppression of tumorigenicity 7
PLA2G12A	1,40	phospholipase A2, group XIIA
PTPLB	1,39	3-hydroxyacyl-CoA dehydratase 2
PIK3AP1	1,39	phosphoinositide-3-kinase adaptor protein 1
TCF7L2	1,39	transcription factor 7 like 2, T cell specific, HMG box
RAB12	1,38	RAB12, member RAS oncogene family
FK21	1,38	feather keratin 21
POLR1D	1,38	polymerase (RNA) I polypeptide D
LDLRAD3	1,37	low density lipoprotein receptor class A domain containing 3
RTN4	1,35	reticulon 4
AP3M1	1,35	adaptor-related protein complex 3, mu 1 subunit
BRD9	1,33	bromodomain containing 9
NDFIP1	1,31	Nedd4 family interacting protein 1
ASPM	1,29	abnormal spindle microtubule assembly
ABCD2	1,28	ATP-binding cassette, sub-family D (ALD), member 2
NR1D2	1,28	nuclear receptor subfamily 1, group D, member 2
CRTAP	1,25	cartilage associated protein
DISP1	0,83	dispatched RND transporter family member 1

Gene symbol	Fold Change	Gene name
SLC6A8	0,81	solute carrier family 6 (neurotransmitter transporter, creatine), member 8
MDFI	0,80	MyoD family inhibitor
NAPRT1	0,79	nicotinate phosphoribosyltransferase
LAMB2	0,75	laminin, beta 2
KLF8	0,75	Kruppel-like factor 8
FLOT2	0,75	flotillin 2
NEK1	0,75	NIMA related kinase 1
RNF166	0,75	ring finger protein 166
IL1RL1	0,74	interleukin 1 receptor-like 1
ACOT11	0,73	acyl-CoA thioesterase 11
ACAD8	0,72	acyl-Coenzyme A dehydrogenase family, member 8
MUC17	0,71	mucin 17, cell surface associated
GPR34	0,71	G protein-coupled receptor 34
GIT2	0,70	GIT ArfGAP 2
ASPG	0,70	asparaginase
DNAH17	0,68	dynein, axonemal, heavy chain 17
SCAF11	0,66	SR-related CTD-associated factor 11
ANGPTL6	0,65	angiopoietin-like 6
CRYBB3	0,64	crystallin, beta B3
SPAG5	0,64	sperm associated antigen 5
COQ10B	0,63	coenzyme Q10B
GNMT	0,63	glycine N-methyltransferase
BTBD1	0,56	BTB domain containing 1
PDIA5	0,53	protein disulfide isomerase family A member 5
MIR144	0,51	microRNA 144
MYO15B	0,48	myosin XV like
YWHAG	0,41	tyrosine 3-monooxygenase/tryptophan 5-monooxygenase activation protein, gamma polypeptide

Table S2. Differentially expressed genes up- and down-regulated in female chickens

Gene symbol	Fold Change	Gene name
TMEM215	6,17	transmembrane protein 215
HSP25	1,98	heat shock protein family B (small) member 9
HSPB11	1,88	heat shock protein family B (small), member 11
IRAK1BP1	1,76	interleukin-1 receptor-associated kinase 1 binding protein 1
APCDD1	1,68	adenomatosis polyposis coli down-regulated 1
SIK1	1,65	salt inducible kinase 1
TCF15	1,64	transcription factor 15
C3orf58	1,62	chromosome 9 C3orf58 homolog
WWC1	1,53	WW, C2 and coiled-coil domain containing 1
TSPAN7	1,53	tetraspanin 7
HSPH1	1,51	heat shock protein family H (Hsp110) member 1
EPB41L5	1,50	erythrocyte membrane protein band 4.1 like 5
ANP32E	1,48	acidic nuclear phosphoprotein 32 family, member E
HSPA8	1,47	heat shock protein 8
CHORDC1	1,47	cysteine and histidine-rich domain containing 1
DDHD2	1,45	DDHD domain containing 2
TJP1	1,45	tight junction protein 1
TMEM62	1,45	transmembrane protein 62
PSTK	1,44	phosphoseryl-tRNA kinase
UBI	1,38	polyubiquitin
CDA	1,36	cytidine deaminase
IGFBP3	1,34	insulin-like growth factor binding protein 3
EFCAB12	1,34	EF-hand calcium binding domain 12
MRPL32	1,32	mitochondrial ribosomal protein L32
PLA2G6	1,32	phospholipase A2, group VI
FAM167B	1,32	family with sequence similarity 167, member B
MAD2L1BP	1,31	MAD2L1 binding protein
CTNNBIP1	1,31	catenin beta interacting protein 1
MRPL18	1,30	mitochondrial ribosomal protein L18
ZSWIM1	1,30	zinc finger SWIM-type containing 1
CEP70	1,27	centrosomal protein 70
CACNA1B	1,26	calcium channel, voltage-dependent, N type, alpha 1B subunit
MEX3A	1,25	mex3 RNA binding family member A
HNRNPA3	0,85	heterogeneous nuclear ribonucleoprotein A3
PDIA6	0,85	protein disulfide isomerase family A member 6
ETFA	0,84	electron transferring flavoprotein, alpha subunit
PPP2R5C	0,81	protein phosphatase 2, regulatory subunit B', gamma
KIAA1715	0,80	lunapark, ER junction formation factor
IDH3B	0,80	isocitrate dehydrogenase 3 (NAD+) beta
OLA1	0,78	Obg-like ATPase 1

Gene symbol	Fold Change	Gene name
BCR	0,78	BCR activator of RhoGEF and GTPase
SLC5A1	0,78	solute carrier family 5 member 1
HLCS	0,77	holocarboxylase synthetase
DARS2	0,76	aspartyl-tRNA synthetase 2 (mitochondrial)
FBXL7	0,76	F-box and leucine-rich repeat protein 7
FAM122A	0,76	family with sequence similarity 122, member A
UBE2T	0,75	ubiquitin-conjugating enzyme E2T
RRAD	0,75	Ras related glycolysis inhibitor and calcium channel regulator
TOX3	0,75	TOX high mobility group box family member 3
SYT16	0,75	synaptotagmin XVI
KIAA0930	0,75	RIKEN cDNA 5031439G07 gene
ZFYVE9	0,75	zinc finger, FYVE domain containing 9
RCSD1	0,75	RCSD domain containing 1
DPF3	0,74	double PHD fingers, family 3
IFITM5	0,73	interferon induced transmembrane protein 5
TG	0,73	thyroglobulin
MICAL3	0,73	microtubule associated monooxygenase, calponin and LIM domain containing 3
RRP15	0,72	ribosomal RNA processing 15 homolog
DYNC2LI1	0,72	dynein cytoplasmic 2 light intermediate chain 1
ZPLD1	0,71	zona pellucida like domain containing 1
WDR25	0,70	WD repeat domain 25
ARHGAP10	0,70	Rho GTPase activating protein 10
MRPS27	0,69	mitochondrial ribosomal protein S27
TRIL	0,69	TLR4 interactor with leucine-rich repeats
COLQ	0,68	collagen-like tail subunit of asymmetric acetylcholinesterase
ZPBP	0,65	zona pellucida binding protein
FGFBP2	0,64	fibroblast growth factor binding protein 2
ZNF407	0,63	zinc finger protein 407
MTSS1L	0,63	MTSS I-BAR domain containing 2
PROM1	0,62	prominin 1
XDH	0,61	xanthine dehydrogenase
TBC1D5	0,60	TBC1 domain family, member 5
NEB	0,60	nebulin
RARRES2	0,59	retinoic acid receptor responder (tazarotene induced) 2
NSG1	0,59	neuronal vesicle trafficking associated 1
FRZB	0,56	frizzled-related protein
PTCD2	0,56	pentatricopeptide repeat domain 2
CTIF	0,54	CBP80/20-dependent translation initiation factor
CES1	0,47	carboxylesterase 1 like 1
LYRM2	0,46	LYR motif containing 2
FAM96B	0,40	family with sequence similarity 96 member B
CEP104	0,37	centrosomal protein 104
PRIM2	0,22	primase (DNA) subunit 2

Table S3. List of genes tested by qPCR

Gene symbol	Chick sex	Upstream Transcription Factor	Pathway	Forward Primers 5'-3'	Reverse Primers 5'-3'	Amplicon size (bp)
ABCD2	Male	MEF2A, NFE2L2	Oxidative stress	CCCATCATCACCGCAACA	TTCACTAACCATTGCCTGCTTCT	72
BRD9	Male	MEF2A, NFE2L2	Oxidative stress	TTCCTTCGTCAGCTTCAAAGG	GGCATCCGTGACTGGAAAAG	63
C1QTNF7	Male	MEF2A, NFE2L2	Oxidative stress	GGGTGTTGCTTTTCCTGGAA	TCAGAGTCCACAGGTTTCTTTACATT	67
DCLK3	Male	MEF2A, NFE2L2	Oxidative stress	GACAATATTTCTGCAGCAGCAAA	TGCCGTGTAGCGCTTCTG	75
KIAA1217	Male	MEF2A, NFE2L2	Oxidative stress	TGCTGGGCAAACAACACAAC	AGCAAGCCCTGTGTTTCGA	70
LDLRAD3	Male	MEF2A, NFE2L2	Oxidative stress	GCCAGATTGCTTCGATGACA	CCACACTTGGACTTGGCTTTC	60
PIK3AP1	Male	MEF2A, NFE2L2	Oxidative stress	AAATCAGCAGTCTGTTGGCTAATG	AATTTTAAAGGCCTGGCACATG	65
PTPLB	Male	MEF2A, NFE2L2	Oxidative stress	CCACGGCCTACCTCGTCAT	CGAACTAGCCCCACAGCAA	73
RTN4	Male	MEF2A, NFE2L2	Oxidative stress	GGGAGTTATCCAGGCAATCCA	AGACTCCAAGTAAGCCCTAAATGG	64
SLC16A10	Male	MEF2A, NFE2L2	Oxidative stress	CCATTGCAGGTTTGCTTCGT	GGGACTCCAGCCAGGTAGAAT	67
TBC1D12	Male	MEF2A, NFE2L2	Oxidative stress	CTATGGTGATTTGGGTCAATGAAA	CTCGAACTCTCCGGGTAGCA	69
ANKH	Male	MEF2A, NFE2L2	Oxidative stress	TATACCCAGCTTTTGACAAGAATAA	TCGCTGTGACGGTGCTATTG	69
ANKIB1	Male	MEF2A, NFE2L2	Oxidative stress	CCGATGACCCCAGTGAGAGT	CACCATGGAAGTGCCATGTC	74
DUS4L	Male	NFE2L2	Oxidative stress	CTGGGCACGTCGTGAAGAT	GGAACGCCAGCTTGGAGTAG	57
HUS1	Male	NFE2L2	Oxidative stress	AAGTTGCCGTGGAGCTACCA	CAGGAATGTCATGCGTCACAA	63
POLR1D	Male	NFE2L2	Oxidative stress	GACCACACGCTTGGCAACT	ACTCCACGTCAGGGTTTTTCA	58
SOST	Male	NFE2L2	Oxidative stress	GACCCTAATGATGTCTCCGACTTC	GGCCCCTCCGTCACGTA	68
STK24	Male	NFE2L2	Oxidative stress	CATCCTGACGCCAAGAGACTT	AGAGCCTCCACCCAGATATTCC	74
ACOT11	Male		Other responsive gene	GGCTGGACCGGAAATACGTT	TTGGTCCCAGGGCACAGA	68
AP3M1	Male		Other responsive gene	GAGCTGATTAAGCCTCCCACAA	CATTACTGCTGCCTGTGATGGA	64
ASPM	Male		Other responsive gene	GTGTGCCAGCGTACAACTCAA	GAAGAGGATGAGGAGGAACAATTTAG	80
BTBD1	Male		Other responsive gene	TCCGAGCAGATAATGCCTTCA	ACTAGCAAGCTGAGGTTCATCAAA	68
MIR144	Male		Other responsive gene	CGCCCTGGGCTGGATATCATC	GCAGCCCAGGGGAGTACATCAT	80
NDFIP1	Male		Other responsive gene	GCAGAGAGAACCAAGGCTGAA	CGTGTCACAAAGTCCTCCTCTCTT	68
OTUD3	Male		Other responsive gene	TTGAACCCTTCGTGGAGGAT	GGCTTTGCCAAATTGGTAACA	61
RAB12	Male		Other responsive gene	TGGACTGTGAAGTTGATCGAGAGA	ACCGCATCCCAGTTATTTGC	75
TPPP	Male		Other responsive gene	GGTGAGCCGGTGCACAA	AGGAGACCTTGGAGGAGTCTTATTT	76
WFS1	Male		Other responsive gene	GTGTTGAGACAGCAGTGTTTTGC	GGAGGATCAGAATTTGAGTTCATTTT	69
INOS	Male		Oxidative stress	CTGACCGTTGCAGTGGTAAACTA	GCAGACTCCATGGTGCAATG	66
SOD3	Male		Oxidative stress	GCCCTGTCTGCCTCTGATGT	AAGTAACGCGCTGCTTGGA	60
PIT 54	Male		Inflammation	CCTGAGCCAGGGCCACTT	GCACACAACTTGAGCATCATTGA	132
COX2	Male		Inflammation	ACCGTAGATGCCCAAGAAGTTG	GCAAGCAGGACTAGGACAATAGC	68
CRP	Male		Inflammation	GCACCGCGCCGATGT	CGGAACACAAACACCTTTCTGTAG	88
MAD2L1	Female	PLAG1	PLAG1 pathway	CGCAATTGTGGTGCAGAATG	TCGGGTTGGCACTTTGTAGTT	57
MEX3A	Female	PLAG1	PLAG1 pathway	AGGAGCCACCATCAAGAGGAT	GGCTGGGCGTGATGATGTA	59
TCF15	Female	PLAG1	PLAG1 pathway	GCGATCTGGACAGCAAACAG	GCTGGTTGCTGAGGCAGAAG	57
TMEM215	Female	PLAG1	PLAG1 pathway	TGGCTCTGGTCAGCGTCTT	ATGCCGGACACGGTGAAC	55
ZSWIM1	Female	PLAG1	PLAG1 pathway	CCAGCCCCTGGCAGTGT	TGGGAGAGTCCTGGACAGAGTAC	59
HSPH1	Female		Other responsive gene	TGCGCGTTCAACAAGAGTAAAC	CCGCCTAGGAAAGGATCAAAT	62
IGFBP3	Female		Other responsive gene	GCTGCAGGAAATTCCAGTGATT	TGGGATGGCCTGATTTTCTAA	69
RARRES2	Female		Other responsive gene	CACCTGGCCATGAAGGACAT	TCCACAGCCCTGCACTCA	56
XDH	Female		Other responsive gene	TCAACAGACTGAAACAGGGATC	CAGCACCTCTTCTACTGAGCTTAAAG	67
INOS	Female		Oxidative stress	CTGACCGTTGCAGTGGTAAACTA	GCAGACTCCATGGTGCAATG	66
SOD3	Female		Oxidative stress	GCCCTGTCTGCCTCTGATGT	AAGTAACGCGCTGCTTGGA	60
SOST	Female		Oxidative stress	GACCCTAATGATGTCTCCGACTTC	GGCCCCTCCGTCACGTA	68
PIT 54	Female		Inflammation	CCTGAGCCAGGGCCACTT	GCACACAACTTGAGCATCATTGA	132
COX2	Female		Inflammation	ACCGTAGATGCCCAAGAAGTTG	GCAAGCAGGACTAGGACAATAGC	68
CRP	Female		Inflammation	GCACCGCGCCGATGT	CGGAACACAAACACCTTTCTGTAG	88
HPRT			Reference gene	GGGATATCGGCCAGACTTTGT	AATCGAGGGCGTATCCAACA	68

Differentially expressed genes up- or down-regulated between Delayed and Control groups from microarray analysis

Genes not tested on the microarray and added to the list
